# Pre-referral rectal artesunate in severe malaria: flawed trial

**DOI:** 10.1186/1745-6215-12-188

**Published:** 2011-08-08

**Authors:** Karim F Hirji, Zulfiqarali G Premji

**Affiliations:** 1 Department of Epidemiology and Biostatistics, Muhimbili University of Health and Allied Sciences, United Nations Road, Dar es Salaam, Tanzania; 2 Department of Parasitology and Medical Entomology, Muhimbili University of Health and Allied Sciences, United Nations Road, Da res Salaam, Tanzania

## Abstract

**Background:**

Immediate injectable treatment is essential for severe malaria. Otherwise, the afflicted risk lifelong impairment or death. In rural areas of Africa and Asia, appropriate care is often miles away. In 2009, Melba Gomes and her colleagues published the findings of a randomized, placebo-controlled trial of rectal artesunate for suspected severe malaria in such remote areas. Enrolling nearly 18,000 cases, the aim was to evaluate whether, as patients were in transit to a health facility, a pre-referral artesunate suppository blocked disease progression sufficiently to reduce these risks. The affirmative findings of this, the only trial on the issue thus far, have led the WHO to endorse rectal artesunate as a pre-referral treatment for severe malaria. In the light of its public health importance and because its scientific quality has not been assessed for a systematic review, our paper provides a detailed evaluation of the design, conduct, analysis, reporting, and practical features of this trial.

**Results:**

We performed a checklist-based and an in-depth evaluation of the trial. The evaluation criteria were based on the CONSORT statement for reporting clinical trials, the clinical trial methodology literature, and practice in malaria research. Our main findings are: The inclusion and exclusion criteria and the sample size justification are not stated. Many clearly ineligible subjects were enrolled. The training of the recruiters does not appear to have been satisfactory. There was excessive between center heterogeneity in design and conduct. Outcome evaluation schedule was not defined, and in practice, became too wide. Large gaps in the collection of key data were evident. Primary endpoints were inconsistently utilized and reported; an overall analysis of the outcomes was not done; analyses of time to event data had major flaws; the stated intent-to-treat analysis excluded a third of the randomized subjects; the design-indicated stratified or multi-variate analysis was not done; many improper subgroups were analyzed in a post-hoc fashion; the analysis and reporting metric was deficient. There are concerns relating to patient welfare at some centers. Exclusion of many cases from data analysis compromised external validity. A bias-controlled reanalysis of available data does not lend support to the conclusions drawn by the authors.

**Conclusions:**

This trial has numerous serious deficiencies in design, implementation, and methods of data analysis. Interpretation and manner of reporting are wanting, and the applicability of the findings is unclear. The trial conduct could have been improved to better protect patient welfare. The totality of these problems make it a flawed study whose conclusions remain subject to appreciable doubt.

## Background

While severe malaria requires urgent attention, patients with the disease often reside far away from health facilities equipped to perform accurate diagnosis and administer suitable parenteral treatment. They may not be able to take oral medication, and face a high risk of permanent disability or death. Can that risk be reduced by an artesunate suppository placed as the patient embarks on the possibly long journey to a clinic? By rapidly lowering parasitemia, rectal artesunate may impede progression of the disease sufficiently to decrease the chance of a grave adverse outcome occasioned by the delay in securing appropriate treatment. Gomes et al. [1] (hereafter referred to without the citation number) report a clinical trial undertaken to test this hypothesis.

Done under the auspices of the WHO and funded by major multilateral agencies, it took place at four centers (one in Bangladesh, one in Ghana, and two in Tanzania). Involving a sizeable team of experts, its design, conduct and analysis were overseen by a renowned clinical trial center. Almost 18,000 people with suspected severe malaria not able to take medication by mouth received an artesunate or a placebo suppository. The outcome was assessed in terms of death by 7 to 30 days (D), or permanent disability (PD).

The main finding was that among the cases who took more than 6 hours to reach a health clinic, *"pre-referral rectal artesunate significantly reduced death or permanent disability (29/1566 [1.9%] vs 57/1519 [3.8%], risk ratio 0.49 [95% CI 0.32-0.72], p = 0.0013)*." The authors unequivocally conclude: "*If patients with severe malaria cannot be treated orally and access to injections will take several hours, a single inexpensive artesunate suppository at the time of referral substantially reduces the risk of death or severe disability*."

This trial is the largest trial ever done for severe malaria, and the only reported trial of this intervention for the noted circumstances. A comment in the same journal lauds it for providing "*clear answers to several questions *..." [2] Two postings on the WHO website give additional information and explanations about the trial [3,4]. Gomes et al. went on to win the prestigious BMJ Group Award for the Research Paper of the Year for 2010 [5]. The accompanying editorial in the *BMJ *described it as a "*remarkable study*" that signified "*an outstanding logistical feat*." [6]

This high profile trial has already had an impact on the global malaria policy. Earlier, supportive data from smaller hospital-based studies had led the World Malaria Report 2008 to recommend rectal artesunate as a pre-referral treatment for severe malaria in children under five [7](page 4). The publication of this trial put that recommendation on a firmer footing, and now it has been broadened to all cases with severe malaria [8](page 3). The principal findings of this trial have been incorporated into other international guidelines for the treatment of malaria, and used to justify pre-referral rectal artesunate as a cost-effective intervention [9,10]. Only one letter to the editor questioning the use of a placebo in the trial broke this mould [11]. For a single trial to have such a recognition and impact, and within such a short time, is a rare event in the modern era.

For trials that address major health issues, it is necessary to check that they were designed and conducted according to required high standards, and their conclusions emanate from a sound interpretation of the findings. The paper by Gomes et al. has not undergone an independent quality review in the context of a systematic review. We thereby undertook to perform an in-depth evaluation of the scientific validity of the trial, and the reliability of its conclusions, and to gauge the contextual ramifications of its design and implementation, including their impact on patient welfare.

## Results and Discussion

We began with an assessment of the quality of Gomes et al. by using an extended version of the Jadad scale [12]. The items in this scale are often employed to assess trial quality in systematic reviews. The seven quality assessment items we used are shown in Table 1.

**Table 1:** Checklist assessment of Gomes et al.

	Trial Feature	Yes/No
1.	Randomization methods described?	Yes
2.	Randomization concealment adequate?	Yes
3.	Blinding level appropriate and adequate?	Yes
4.	Patient flow chart given?	Yes
5.	Attrition bias low or not present?*	Yes
6.	Intent-to-treat analysis used?*	Yes
7.	Overall drop out level ≤ 10%?*	Yes

Total Score (Yes = 1, No = 0; Maximum = 7)		7

Note: Items in the checklist based on Jadad and Enkin [12] and the CONSORT statement [13]; *As stated by the authors.

Our subsequent detailed assessment was performed along five principal lines: (i) trial design; (ii) trial conduct; (iii) data analysis; (iv) interpretation; and (v) contextual issues including patient welfare. In this task, we used generally accepted quality criteria for the internal validity of clinical trials in the literature and as reflected in the CONSORT Statement [13,14]. Trial features that pertain to external validity were also examined [15-17]. Where possible, reporting quality was separated from substantive quality. Other than this, we did not have a formal evaluation scheme. We present our findings as a narrative-based review in which we lay out the evidence and line of reasoning we employed to reach our conclusions.

Where needed and if feasible, we reanalyzed the trial data using either the methods used by the authors, or different methods, as implemented in the package WINPEPI [18]. The specific methods are noted in the relevant sections. All unsourced quotes in this paper are from Gomes et al. In addition to the WHO website postings noted above, two papers that either draw upon the original trial data, or concurrently interviewed a subset of trial participants, provided additional relevant information. The first paper, Kitua et al. [19], examines the ethics of using a placebo in this trial, and the second, Gomes et al. [20], reports a parallel investigation done at one trial center. The principal author and several co-authors of each paper participated in the trial.

### Synopsis

This trial was double blind with a treatment allocation scheme that was random and adequately concealed. Only eight cases were said to be completely lost to follow-up. The data analysis is stated to have followed the intent-to-treat principle. According to the items in Table 1 and taking the descriptions given by the authors at face value, this trial secures a perfect score, implying that it is of high quality. On the other hand, our assessments revealed that it had major flaws in all the five main facets we looked at. We detail these findings below.

### Trial Design

A child in a remote village has high fever, and may be vomiting, convulsing, or comatose. The signs indicate severe malaria but that cannot be confirmed. A pill by mouth is not feasible. Injectable drugs are unavailable. Should an artesunate suppository be placed before the child is sent to a health clinic where the required diagnostic tools and treatment are available? That is the basic question Gomes et al. tackle.

Half of the randomized cases in this four-center study were in Africa, and half in Bangladesh. All centers used identical active and placebo treatments, and the randomization scheme was stratified by center and blocked. A core common design was developed, and was subsequently "*adapted*" for each country.

Between center differences in terms of disease characteristics and baseline risk are, to a degree, inevitable in a multi-center trial. For example, the Bangladesh sites were in low malaria transmission localities but at the African sites, the levels of transmission were high. The parasite subtype distributions in Bangladesh and Africa differed. The hallmark of a multi-center trial, nevertheless, is commonality of basic design features across the centers. When applied appropriately, this design can provide better insight into the applicability of the findings of the study.

In this trial, a number of features that should have been similar across the centers were not. For example: African centers enrolled children up to the age of seventy-two months but in Bangladesh, older children and adults were recruited as well. The cases in the latter had free hospital and supportive care. In Africa nothing like that was arranged. At the referral clinic in Bangladesh, intravenous quinine was given. In Africa, intramuscular injections were given. There were about two recruiters per village, on average, in Africa; in Bangladesh, there was exactly one recruiter per village. The calibre of the recruiters, and the training schemes perhaps differed. One blood slide per case was taken in Africa but two were taken in Bangladesh. The types of data collected varied somewhat. The African centers themselves had differences in design. We give more details later. These differences put this trial in the grey-zone between a well designed multi-center trial, and a collection of two, three or four distinct trials with their own protocols. This fact has profound implications for data analysis, interpretation of the findings, and future meta-analysis. We elaborate on these points later.

The tested treatments, randomization scheme, and settings are adequately described in the paper, as required by the CONSORT statement [13]. Two outcomes, death and PD, are declared as the primary endpoints (main outcomes). A number of key items, however, are partly specified or missing. The omissions cast a shadow on what actually happened. These items are (i) sample size computation; (ii) eligibility criteria; (iii) nature of training for trial recruiters; (iv) the time plan for follow-up of the subjects; and (v) quality assurance for blood slide readings.

The absence of sample size computation information in a trial guided by a prominent clinical trial center may be a reporting oversight. We also do not know the basis on which the total size was apportioned across the centers. Such information not only tells us if the trial had high power to detect realistic differences but also assists us, in the absence of protocol deviations, to judge the assumptions made, identify primary outcome(s), effect measure, relevant data analysis method, and the levels of loss to follow-up and missing data [21].

As the analysis employed three main outcome variables (D, PD, and D or PD), we used a Bonferroni adjusted *α *= 005/3 = 0.0167 to compute the sample size needed in a two group design to detect a difference in an outcome of 1.0% and 2.0% with 90% power. This turned out to be *n *= 8000. The trial size thus sufficed to detect such a difference. But, our computation assumed negligible between center heterogeneity, which was not the case. Extensive post-hoc subsidiary analyses, with some being accorded primary importance, were also done. That increases the *α*-error making the statistical significance of any comparison difficult to judge.

A clear declaration of the inclusion and exclusion criteria is an essential feature of trial reports [13,14]. Gomes et al. state in several places (but in not quite consistent ways) that they recruited cases with symptoms of severe malaria who could not take oral drugs and the methods section mentions recruitment of "*patients meeting eligibility criteria*." But the signs and symptoms the recruiters were trained to identify and use for inclusion and exclusion are nowhere explicitly noted, and center or country-wise differences in these criteria are not mentioned. That the age ranges for the three countries were not the same indicates that each had its own criteria. Also, the list of inclusion but not the exclusion criteria at one Tanzania center for this trial appear in another paper published a year later [20].

Trials in rural settings may not have the elaborate eligibility criteria of hospital-based trials. Yet, they do need some criteria, however rudimentary. And, even these need to be stated clearly and unambiguously.

The study population is not described consistently. The Methods part in the Summary says that "*patients with suspected severe malaria ...*." were recruited, but the Interpretation part gives recommendations for "*patients with severe malaria ...*" Another paper derived from this trial states that the "*study population comprised patients with suspected severe malaria ...*." but then reports data analysis for only those with confirmed malaria [19].

The type of recruiters deployed and their training is a critical and related issue. Rural trials often employ community health workers, nursing assistants, medical assistants, local midwives, medicine sellers or even traditional healers in that role. These village health workers (VHW) undergo further training on diagnosis, enrollment decisions, drawing blood samples, administering treatment, maintaining basic records and performing other tasks. For example, in a trial of intra-rectal quinine alkaloids for severe malaria, Thera et al. [22] involved employees at rural health facilities who underwent additional training, the aims and content of which are clearly stated. In a study of home management of malaria, Ajayi et al., [23] used community medicine dispensers and field supervisors. All attended workshops to learn specifically noted techniques and matters. The former also underwent a refresher course one month after initial training.

The key point is to use people already immersed in the provision of health care at some level, and give them suitable training, the nature and duration of which are well described either in the report, or in a referenced document. An example of the latter is WHO [24]. The more serious the disease, the higher are the standards for trainees and training that need to be adopted.

Gomes et al. does not compare well with the above noted trials in this regard. They used 417 "*resident village recruiters*," most of whom had "*little previous medical knowledge and no research experience*," who then "*underwent one to three weeks of training*." [1,3] The kind of training given is not described in the paper nor the website material. It is not clear why some were trained for a week and others for three weeks, and whether the calibre of the trainee recruiters and the training protocol varied by center or not. The recruiters are stated to have been supervised every few days by a team of 74 field supervisors named at the end of the paper. But their qualifications, and the additional training they received, if any, are not described [1,3].

The WHO website material is inconsistent with the paper. The website describes the recruiters slightly differently as persons with "*little or no previous medical knowledge ..*" and are inaccurately referred to as "*village health workers*." [3] A related paper calls them "*semi-trained village health worker[s]*" in one place and "*community health worker[s]*" in another, but does not spell out what the terms mean [20]. Kitua et al. [19] says that in this trial "*community-based recruiters were trained to identify patients (with clinically suspected malaria, who could not take oral medication) early in the evolution of the disease, to randomise them to a single dose rectal artesunate or identical placebo, and immediately refer each patient to medical facility ...*" But, the nature and duration of the training, or whether the recruiters were trained to draw blood, make blood slides, note date of birth and sex, and accurately fill out the entry form and referral slip are not mentioned.

The information about training and trainees in this trial is scanty, not always consistent, and scattered in several papers and sources. It requires a resourceful reader to unearth them. The phrase "community" or "village health worker" has an official definition that varies in different national contexts [25]. Lack of clear terminology on such matters impedes appropriate interpretation and practical translation of research findings.

Another design-related concern is the time window for initial follow up. Most severe malaria studies assess short term or in-hospital mortality (see Table one in Maitland [26]). Akech et al., [27] however, followed up severe malaria cases after discharge. Of the 241 patients enrolled, 213 were discharged alive. By tracking them through an established demographic surveillance program, 196 were confirmed alive in the 21 to 35 day period after discharge. The short term (in-hospital) mortality here was 11.2% and cumulative medium term (up to 35 days) mortality was 18.3%. The cohort study of Phiri et al. [28] also indicates continued higher post-discharge risk of death among severe malaria cases.

The first follow-up window in Gomes et al. trial is stated as 7 to 30 days after entry. The first follow-up "*took place, on average, at day 14*." Even while it refers to curves that look like survival curves (Figure two of Gomes et al.), it is unclear if this is a median or mean value. No other details like median follow-up time by treatment arm or center are provided. In any case, it implies that some patients were possibly first followed up on day 8, some on day 29, etc. Those followed up early were assessed for short term mortality, while those followed up towards the end of the period, for cumulative medium term mortality. This obscures clinical interpretation of the results as apples are mixed with oranges. Also we do not know whether the follow-up window was set by design, or if it was a *post hoc *reflection of what occurred in practice. The wide follow-up window also compromised the data analytic strategy used (see below).

Quality control for blood slides is not mentioned. Confirmatory diagnosis is essential in severe malaria. Blood slides results need quality control [29,30]. During the parasite sequestration stage, moreover, peripheral parasite density in severe malaria can be low or even negative.

For each issue noted above, the extent of between center variation bears on the interpretation of the results. Yet, hardly any relevant details are given.

The example of Yeboah-Antwi et al. [31] is useful to obtain a comparative perspective. This paper reports a trial of community management of fever in rural Zambia. With a cluster randomized design and sample size of 3125 children, the aim was to see if VHWs could be trained to distinguish between and treat uncomplicated malaria and non-severe pneumonia effectively.

In Table 2, we compare the adequacy of reporting of design features for this trial with that of Gomes et al. Unlike the latter, the Zambia trial report gives sufficient details on sample size calculations, eligibility criteria, and prespecified primary and secondary outcomes. The background of the VHWs, and the methods, content, type and duration of training given to them, and the skills assessment and supervision done are described in depth. The planned follow-up window is precisely stated. The trial protocol and training manuals are posted on the journal website as supplementary files [31]. While this report stands at the high end of the good reporting scale, and most rural trial reports would fall somewhere in between, our impression is that Gomes et al. would fall below the half-way level on that scale.

**Table 2:** Adequacy of the reporting of trial design features

Feature	Reported and adequately described?	
	**Gomes et al. **[1]	**Yeboah-Antwi et al. **[31]

Protocol Publicly Available	No	Yes
Study Question	Yes	Yes
Study Population	Inconsistent	Yes
Basic Study Design	Yes	Yes
Treatment and Control	Yes	Yes
Inclusion Criteria	Vague	Yes
Exclusion Criteria	No	Yes
Primary Outcome(s)	Unclear	Yes
Secondary Outcomes	Unclear	Yes
Randomization Scheme	Yes	Yes
Blinding Level	Yes	Yes
Evaluation Schedule	No	Yes
Sample Size Computation	No	Yes
Statistical Methods	Partial	Yes
Background of Recruiters	Vague	Yes
Type and Duration of Training	Vague	Yes
Training Manual	No	Yes
Skills Assessment	No	Yes
Trained Data Collectors	No	Yes
Supervision	Yes	Yes
Informed Consent	Yes	Yes

### Implementation

An apparently positive feature of the trial was that only eight out of the nearly 18,000 subjects were completely lost to follow-up. We say apparently because first, the follow-up schedules set in the trial protocols are not known, and second, what under one way of analyzing the data looks as a high loss to follow-up level can, under another way of analyzing them, become a low loss to follow-up level. This point is elaborated in the next section.

There were two other major implementation-related shortcomings, namely, (i) recruitment of clearly ineligible subjects, and (ii) failure to collect key data for a large number of subjects.

According to Figure one of the paper, the randomized children fell into three subgroups (i) cases negative for malaria (*n *= 4648), (ii) cases already treated by injection for severe malaria (*n *= 1110), and (iii) subsequently confirmed malaria cases possibly needing injectable treatment (*n *= 12068).

The randomization (enrollment) form for the trial recorded whether the subject had had an immediate prior injection for malaria or not. Cases in subgroup (ii), 6% of the total, were thus identifiable but clearly ineligible for this trial. Why did the recruiters randomize such cases? Did this anomaly occur only at some centers? What does it say about the training process? The authors implicitly, after the fact, and without a clear explanation, acknowledge the ineligibility of such subjects by excluding them from all data analysis.

The number of cases in subgroup (i), almost 25% of the total, is a related issue. Some possibly were false negatives as peripheral parasite density in severe malaria is negative in the sequestration phase. But presumably most had pneumonia, meningitis or another infection. Properly trained VHWs can differentiate between malaria and pneumonia, and reduce child mortality from these conditions [31,32]. A rapid diagnostic test for malaria, which a VHW can be trained to use efficiently, assists in this task [31,33]. Cheaper brands of such tests were becoming available at the time this trial began. At the community level, misdiagnoses of malaria and severe malaria are inevitable. The point is to minimize erroneous diagnoses with adequately trained VHWs, and perhaps a rapid diagnostic test. The training done by Yeboah-Antwi et al. [31] is exemplary towards achieving such a goal.

Now consider the issue of missing data, starting with age. Table one of the Gomes et al., which reports baseline comparisons, shows the age distributions by treatment group in an incomplete way. Only subjects older than 6 years for Bangladesh are fully compared. The legend of the table refers to "*apparent age*," and in the text, there is reference to "*assessed age*." (paragraph 1, Results section). These terms are never defined, and we are not told who did that assessment, and how it was done. Another concern is that the "overall mean age" for children in the artesunate group is given but is not given for those in the placebo group, and the age distributions by treatment group for children are not given. Also, while the centers in Tanzania recruited children with "*assessed age*" between 6 to 60 months, the Ghana center recruited children with "*assessed age*" from 6 to 72 months. Why the difference? The bottom line is that baseline comparison for a basic item like age is not fully and clearly reported. Perhaps that was due to extensive missing data on age. It seems that the problem was more serious in the African centers.

Next, we note that at Handeni, a Tanzanian center, "*blood slides were not collected routinely during most of the trial*." (Legend to Table one of Gomes et al.) In all, about 17% of the cases included in the analysis from Africa had no blood slide. For Bangladesh, this stood at less than 1%. How was it determined if a case with no slide actually had malaria? How were such cases included in the analysis? No clear answers to these critical questions are provided.

This problem is related to the earlier observation that cases in Bangladesh had two slides taken at enrollment while those in Africa had only one. At all sites, one slide was retained with the enrollment form for collection by trial personnel. The extra slide in Bangladesh was taken by the patient, together with the referral slip, to the referral health facility, and used, perhaps with an additional newly made slide, for diagnosis. In Africa, a pre-referral slide was not available at the health facility [20]. Since rectal artesunate rapidly lowers the peripheral blood parasite count, the sole reliance on the slide taken at health facility raised the chance of a false negative in the artesunate arm compared to the placebo in Africa. Therefore, an African case with severe malaria in the artesunate arm had a higher chance of delayed treatment for severe malaria, thus affecting outcome and the internal validity of the trial.

Another item with many missing values is time to reach the clinic. This is a critical data item for this paper, in that apart from Table one and Figure one, all the other tables and figures in the paper (Tables two, three and four and Figures two, three and four) relate to it. Most of the analyses in the Results section pertain to it. And it is these analyses that generate the conclusion that the real benefits of pre-referral rectal artesunate for severe malaria become evident only when we account for time to arrival at the referral clinic.

Yet, the Legend to Table three states that time to clinic "*was recorded routinely only in Kilosa and Navrong*." That is, only two African sites, with fewer than half of the eligible randomized subjects, regularly gathered data for a variable which underpins the bulk of the analysis done. To make up for this shortfall, the analysis **made a reasonable guess **for the value of this variable for a subject with a missing value. The authors **assumed **that 95% of the cases reached the referral clinic within six hours, and 50% of the cases at Handeni, the third African site, were **assumed **to have reached the clinic within this window.

The favorable arrangements under which most patients were expected to, and promptly did, go to a hospital in Bangladesh may explain why it was not in the routine data collection plan. Thereby, usage of the variable "time to clinic" in the analysis was a post-hoc decision. The bulk of the analysis presented, and which lead to the principal recommendations of the trial, is thus seen not to derive from field data but from not well justified imputations for a crucial data item for up to 50% of all the subjects used in the analysis.

Even where it was recorded, we do not know who recorded the time to clinic, and how precisely it was defined. For example, in Kilosa, patients often first went to a smaller health clinic they were referred to by the recruiter and later landed in the main district hospital where the required or further treatment was given. Gomes et al. define it as "*time to reach a facility at which injections could be given*" while a related paper with the same main author specifically deals with time to reach the district hospital [20].

The recruitment of clearly ineligible subjects, the missing slides, problems of data collection and the concerns about the quality of some data items do not reflect positively on the training and supervision at the African (especially Tanzanian) sites, and the types of recruiters they used.

#### Conflicting Descriptions

Gomes et al. [20] report a parallel study done at Kilosa, a trial center in Tanzania, whose aim was to assess the impact of the referral advice given to the trial participants. The authors interviewed the guardians or parents of 936 children admitted at Kilosa district hospital during the time the trial was underway. Of these children, 880 were enrolled in the trial and 156 were regular admissions. Of interest is the following statement. "*Patients were assessed for admission by the admitting clinical officers who were unaware of whether patients were included in the community-based study*." [20] The community based study in question is the trial of Gomes et al.

Kitua et al. [19] provide a detailed justification of the ethics of using a placebo in the Gomes et al. trial. This paper stresses that protecting the welfare of the patients was a major consideration in the trial. Arrangements were made to enhance adherence to referral advice, and assure appropriate care once the patient arrived at a health facility. We leave some aspects of this matter for later discussion. Of interest for now is the following statement. "*In Tanzania, patients were provided with a referral slip identifying them as a study child, their entitlement to free hospital care was reinforced*." [19]

If specific steps were indeed taken to identify the trial participants so as to assure them free quality care, it is difficult to see how the blinding in terms of trial participation that Gomes et al. [20] note could have been enforced. Hence the situation in this regard is unknown What Kitua et al. [19] say is in line with the main report, and casts a negative light on the validity of the parallel study of Gomes et al. [20]. We note that the main author of each of these papers is a co-author on the other paper, and both are co-authors of the main trial paper.

These conflicting statements about an important feature of the trial from the trial investigators together with the other incomplete, vague or inconsistent descriptions noted elsewhere reinforce the impression that the reportage in Gomes et al. is substantially deficient. An independent audit of trial records may be needed to clearly establish what transpired during the course of the trial.

### Analysis and Interpretation

The data analyses presented in Gomes et al. are seriously deficient in eight ways. These are: (i) ambiguous primary end points; (ii) incorrect analyses of time to event data; (iii) flawed intent-to-treat analysis; (iv) absence of an overall analysis; (v) absence of design indicated stratified or multi-variate analysis; (vi) analysis of improper subgroups; (vii) excessive post-hoc analyses; and (viii) the use of a less than desirable analysis and reporting metric.

Before we elaborate, we reiterate an observation made earlier, that the extent of center-specific variability places this study somewhere between a bona-fide multi-center trial, and two or three or four separate trials. The authors faced three basic options for the main analysis of the data. One, analyze and report as separate trials. Two, analyze as for a multi-center trial employing methods for stratified data. And three, just add the numbers from all centers, and analyze as for a single trial with a uniform protocol. The extent of design-based heterogeneity, and the paradoxes associated with it, make the last option the least advisable [34]. But it was the option selected by Gomes et al. This affects both the interpretation of the findings, and the conduct of future systematic reviews. We elaborate on this when we discuss the issue of stratified analysis below.

#### Ambiguous Primary End Points

The primary endpoint in a clinical trial is specified in the trial protocol, used in sample size computation at the planning stage, and, once the trial is over, is used in the main data analysis and for reporting the results in the abstract. It should be clearly identified as such in the methods section of the report. There may be more than one primary end point, but trials with more than three primary endpoints are rare. The terms primary outcome and main outcome are synonyms. One primary end point then corresponds to one outcome variable in the analysis.

Gomes et al. clearly state, with different wordings, in three places (Summary-Methods; Methods-Outcomes; Methods-Statistical Methods) that the study had two primary endpoints, namely 7-30 day mortality and permanent disability. However, in the Results section, we read that "*... the main analyses are of death or permanent disability for 12068 patients with malaria ...*." The two primary endpoints thereby produce a third composite endpoint that goes on to supersede them in importance. The bulk of the results presented in the Results section of the Summary are in terms of this composite outcome variable. And, by that point, the term mortality has been reinterpreted as late mortality (death after six hours).

#### Event or Time to Event?

The principal formal data analyses in Gomes et al. are in terms of proportions. In a follow-up design, computing a proportion assumes a fixed time window. Data analysis for an uncomplicated malaria trial, for example, usually evaluates a 14-day or 28-day treatment failure rate. Minor variation in follow-up times, as in a window of 13-15 days for the 14-day rate, is inevitable and acceptable. But if, as judged by the nature of the disease, times to follow-up vary extensively, the appropriate course of action is to analyze the data as time to event data.

Accordingly, Figure two of Gomes et al. shows the cumulative death rate, and the cumulative D or PD rate over time (by continent and treatment). The method used for estimating the curves is not stated. Their first portions (up to 7 days) treat the data appropriately as time to event data. But in the second portions, the time window is compressed, and simple proportions are given. By presenting these as survival curves but not exactly declaring them to be as such reduces clarity. A reader may take all the proportions as cumulative proportions, and the last ones can be misinterpreted as the 30 day death (or D or PD) rates.

Take the outcome death. Three data items were available for each case: the day of the first follow-up visit, the status of the patient on that day (dead or alive), and if dead, the day of death. For those who died before day 7, either that fact became known at the time, or at a subsequent follow-up visit. Since, except for the very few cases with suspected PD, no further follow-up occurred, the data are censored data that are usually analyzed with survival data methods, or as person-day data. Even if the main analyses were done in terms of simple proportions, the authors could have shown the appropriate cumulative proportions to day 30 in Figure two with the Kaplan-Meier method. Appreciable variations in the follow-up times generally, by treatment arm or by center can produce different results from different methods of analysis.

Take a simple example: Suppose there were four cases in each group, and the planned follow up was at 30 days after entry. But, poor implementation caused it to be done on an ad hoc basis. The respective days to death data for the artesunate and placebo groups were: {8+, 9+, 27, 28} and {8, 9, 27+, 28+}. Here, '+' represents a censored observation. Under the planned binary outcome analysis of the 30 day survival rate, follow-up at 27 or 28 days may be deemed close enough and acceptable. But the censored status of the first two cases in the artesunate group is so far from the target day that their final outcome would have to be regarded as unknown, and their data treated as missing. To use these early censored data, a survival analysis is needed. Instead, Gomes et al. went ahead with the proportions-based analysis but now under an expanded time window of 7-30 days. This not only mixes up short term and medium term mortality but also makes the loss to follow-up rate appear lower than it really was. In this example, the wide window makes the missing data rate drop from 25% to 0%.

The wide window can also produce misleading results. Under their analysis plan, the 7-30 day death rate is 50% for both groups. Now suppose that the actual status of the cases 30 days after entry was given by {24, 30+, 27, 28} and {8, 9, 30+, 30+}. The actual 30 day death rate for artesunate is 75% and for placebo, it is 50%.

Note, if among those who did not experience an event, a fixed minimum time of follow up was recorded, it is legitimate to do both binary and survival types of analysis. This was done, for example, for a community-based diarrhea trial [35].

From a clinical perspective, the wide time window in Gomes et al. is not useful or realistic. Together with the binary analysis approach, it can mask the true rate of missing data and yield conclusions that deviate from underlying reality. Conceptually, continued medium term risk of death makes a 7-30 day death rate for severe malaria as valid as a 2-5 year death rate for a cancer study.

#### Intent-to-Treat Analysis

Data analysis in a clinical trial should not be restricted to the cases treated under ideal clinical conditions, who behave as perfect cases and whose status is know at all time points. Removing randomized cases from analysis for violating such conditions not only affects the applicability of the findings but also biases the estimate of treatment effect [36]. To address these concerns, an intent-to-treat (ITT) analysis (i) includes all randomized subjects, and (ii) places each subject in the group to which he or she was randomized [13,37,38]. This is done even if the case did not actually get the allocated therapy, switched therapy, failed to take the full dose, was misdiagnosed, should not have been in the trial, was lost to follow-up, or underwent any other experience. Subjects whose final outcomes are not known are often assigned values that least favor the adoption of the new treatment. The ITT approach recognizes that treatment anomalies reflect real life, protects the control of bias attained by randomization, and reflects a precautionary approach towards using the new intervention.

Gomes et al. included eight cases in whom the inserted capsule was almost immediately expelled in the analysis. Also, eight cases completely lost to follow-up were included on the assumption that they all recovered. Thus, they declare their analyses to be ITT analyses. Yet, they excluded all the subjects in subgroups (i) and (ii) from all analyses. They justify this act by stating that the exclusion decision was taken prior to breaking the blinding scheme. Nonetheless, removing a third of the randomized cases from all the analyses is a major violation of the ITT principle. If a typical malaria trial has about 500 subjects, the nearly 6000 randomized subjects excluded from analysis is equivalent to dumping data from 12 trials!

Excluding the cases with a prior injection from data analysis represents, as noted, a modification for a design or implementation-related flaw. Excluding patients with negative slides, however, lacks a conceptual or practical justification. The remote areas where the trial was conducted and where its results would apply are areas where confirmatory diagnosis of malaria or severe malaria cannot be done. Whether in a trial or in practice, presumptive treatment prevails. Even with well trained VHWs and a rapid diagnostic test, cases given a suppository would later be found not to have malaria, and some with malaria would be missed, especially if they have a concurrent infection.

We performed a true ITT analysis for this trial by including all cases as randomized. The data for this exercise were extracted from Figure one of the paper (see Table 3). For the eight lost subjects, we posited three scenarios. Scenario I assumed, as done by the authors, that they were alive without PD. Scenario II assumed they were dead, and scenario III assumed that they were alive but with PD. The overall treatment effect *p*-values for scenarios I, II and III are 0.18, 0.11, and 0.027, respectively. The close follow-up for cases with possible PD makes scenario III the least likely of the three. And, even for scenario III, the *p*-values for the individual outcomes are not significant at the 0.05 level when adjusted for multiplicity. An unbiased ITT analysis with higher power thereby does not provide sufficient evidence for a treatment-related difference for the main outcomes. However, even these ITT analysis are marred by the data quality and time window concerns raised earlier.

**Table 3:** ITT analyses under three scenarios

Status	Scenario I		Scenario II		Scenario III	
	
	Artesunate	Placebo	Artesunate	Placebo	Artesunate	Placebo
Dead*	258	288	258	296	258	288
Alive*& PD	15	22	15	22	15	30
Alive*& No PD	8681	8562	8681	8554	8681	8554

Total	8954	8872	8954	8872	8954	8872

*p*-value**	0.18		0.11		0.027	

Note: These analyses include all the cases as randomized and use the data Figure one of Gomes et al. Scenario I assumes that the eight missing cases were alive and without PD, scenario II assumes they were dead, and scenario III assumes they were alive but with PD; *Dead or Alive by day of 7-30 day follow up; **Chi-square *p*-value.

We further discuss the authors' justification for excluding one third of the randomized subjects from the data analysis when we address the practical concerns related to this trial.

#### Overall Analysis

After excluding cases from subgroups (i) and (ii), the authors are left with the following results: artesunate (D = 154, PD = 2, Total = 6072), and placebo (D = 177, PD = 12, Total = 5996). Even though they had two primary endpoints (D and PD), they analyze these data in terms of three binary outcome variables (D, PD, and D or PD) and find that the *p*-value for D is not significant (0.1); for PD, it is highly significant (0.002), and for D or PD, it is at a borderline level of significance (0.048). The observed difference in mortality is thus not statistically significant when considered by itself but becomes barely significant when it is combined with PD.

We avoid this quandary if we start with a combined analysis of the outcomes and then, for separate outcome comparisons, we adjust the *p*-values for multiplicity [39]. This approach is consistent with a joint analysis of the two original primary endpoints of the trial. It is the approach we used in the ITT analyses above. For these non-ITT data, using a three-valued outcome variable (D, alive and PD, alive and no PD) gives a chi-square *p*-value of 0.006, signaling the presence of an effect beyond chance variation. Further, making separate comparisons under adjustments for multiplicity, we find that only the difference in PD rates is significant.

The declaration of two primary end-points in the Methods section indicates that the third composite outcome variable (D or PD) was chosen post-hoc. Further, no time factor is attached to the variable PD, making its composition with D conceptually problematic. In the Results section, primary and secondary outcome variables are not clearly distinguished. Also, note that even the overall analysis we give is suspect because of the wide window; because it is, like the authors' analysis, not an ITT analysis; and because it has not been done in a stratified manner (see below).

#### Stratified Analysis

The randomization scheme and design-related heterogeneity between the centers call for a stratified form of analysis. Figure two of Gomes et al., for instance, points to a continent-wise heterogeneity of effect. Such an analysis would, to an extent, compensate for not publishing as separate trials. Stratification allows us to adjust for the differences in baseline risk and design factors into the analysis. The main analyses done by the authors, however, simply added the numbers from all the centers.

Because center-specific data for subgroups (i) and (ii) are not given, an ITT stratified analysis cannot be done. In that case, using the same data as used by the authors gives a comparative perspective. Table 4 has the data on D or PD rates by treatment stratified by center. For each center, the 95% CI for the risk difference (RD) for D or PD includes the null value. We fitted a random effects model [40] to estimate the overall RD. This model allows for between strata heterogeneity by positing the stratum effect as a normally distributed random variable with unknown but constant variance. The resultant estimate of the RD was 0.006 with 95% CI (-0.003,0.014). This result is not consistent with the findings of the authors.

**Table 4:** Risk of death or PD by center and treatment

Center	Artesunate (%)	Placebo (%)	RD (95% CI)
Africa-Handeni	54/726 (7.4%)	71/737 (9.6%)	2.2% (-0.8%, 5.2%)
Africa-Kilosa	27/1170 (2.3%)	31/1169 (2.7%)	0.3% (-1.0%, 1.7%)
Africa-Navrong	30/1145 (2.6%)	43/1093 (3.9%)	1.3% (-0.3%, 2.9%)
Bangladesh	45/3031 (1.5%)	45/2997 (1.5%)	0.1% (-0.6%, 0.7%)

Overall	156/6072 (2.6%)	190/5996 (3.2%)	0.6% (-0.3%, 1.4%) *

Note: Due to lack of relevant data, these analyses are not ITT analysis. Instead, they use the subjects analyzed by Gomes et al. with death assessed by the day of the 7-30 day follow up; *Random effects model estimates.

While the main analyses of Gomes et al. were not stratified, some subsidiary analyses were. Thus, the proportions who never reached the clinic among those who survived more than 6 hours were analyzed, in a stratified manner, by study center, and showed a marginal effect. The reasons for doing a stratified analysis here but not elsewhere are not given.

Reporting this study as a single trial without sufficient strata level details bears on the conduct of a future systematic review. The level of heterogeneity may prompt the reviewers to treat it as two, three or four separate trials, as was done, for example, in a meta-analysis of treatments for diarrhea [41]. But Gomes at al. does not provide the basic data required for such a task. For example, the numbers randomized to placebo and artesunate are not given by continent or center.

#### Data Dredging and Subgroup Analysis

Analyzing data restricted to a subgroup of the study subjects is called subgroup analysis. The medical literature abounds with warnings about the pitfalls of such analyses [42-51]. Subgroup analyses are frequently overdone, done post-hoc, and performed inappropriately, and as such, raise the chances of producing flawed, false positive conclusions.

The primary analyses of Gomes et al., which exclude a third of the randomized cases, are, to begin with, subgroup analyses. The greater portion of the additional analyses further divides this subgroup into smaller subsubgroups. The division used most often separates those who died within six hours and those who did not. Most analyses exclude the former, as is evident from Tables two, three and four, Figures three, four and five of Gomes et al., and the amount of text devoted to these tables and figures. The latter subsubgroup is decomposed into smaller and smaller entities (by region, time to reach the clinic, age, comatose or not, etc.) for treatment-wise comparison. For example, the treatment-wise comparison of resolution of CNS damage giving *p *= 0.0037 employed only 44 of the 17,826 randomized cases. As data-driven analyses are often reported selectively, it is safe to say that the total number of such analyses the authors did probably exceed those reported in the paper.

None of the multitude of *p*-values is adjusted for multiplicity, and are difficult to interpret [52,53]. The authors state (in the Discussion) that the "*main finding *[of the paper] *is based only on 3000 of the 18000 patients originally recruited ..*" Most of the results in the Summary also derive from analyses of subgroups constructed within other subgroups.

Was subgroup analysis of any form pre-planned? The authors say that "*the cutoff of 6 h in our analyses was not prespecified ...*" The bulk of the analyses uses this cutoff. Since the data on time to reach the clinic were not routinely collected at all centers, it is unlikely that time to clinic-based analysis of any form was pre-specified. Unlike some other analytic decisions, we are not told whether the use of the 6 hour cutoff was decided prior to breaking the blinding code or not. It is not as well explained why a 6 hour, rather than, say, a 12 hour cutoff, was selected.

A serious concern is that most of the subgroup analyses in the paper are in fact the most proscribed forms of subgroup analyses. To grasp this point, note that subgroups are of two types: those defined by baseline features, and those defined by an event or feature that is manifested after randomization. Analysis of the former is acceptable if it was specified in the protocol and uses an valid interaction test. Analysis of the latter, called improper subgroups, is, however, discouraged under any circumstance [43,51,54-56]. Analysis of improper subgroups can mislead even when baseline balance prevails. Subtle interactive effects can make treatment arms within improper subgroups different in terms of important prognostic features (baseline and time based), no longer directly comparable, and make this form of analysis prone to time-dependent bias [57,58].

The bulk of the statistical analysis in Gomes et al. divides the patients in terms of a post-randomization event, namely, time to death. In Table 2 and later analyses, one of these subgroups is further decomposed in terms of time to reach the clinic (0 to 6 hours or more than 6 hours). Improper subgroups are formed within improper subgroups and are again divided. The validity of these analyses is methodologically suspect. The manner in which the division is done also fosters conceptual confusion. For example, cases who arrived at the clinic within 6 hours but died within 6 hours, or those who died within 6 hours but were on their way to the clinic disappear from view. On top of that, all detailed analyses of time to reach the clinic are, as noted earlier, not based on a firm foundation in that this time is not well defined and was routinely recorded at only two of the four participating centers.

If such practical problems are not present, is there a better method to analyze the data? Using the data from Figure one and Table two of Gomes et al., we show in Table 5 how time to death can be incorporated into the analysis in an unbiased manner that also reduces the chance of false positive findings. Here, the final status of each patient is classified into one of four categories (dead within 6 hours, died after 6 hours; alive but with PD; alive without PD). We compare the treatments for this four category outcome variable by an overall chi-square test. We also perform individual category comparisons, adjusting the *p*-values for multiple comparisons.

**Table 5:** Early death, late death or PD by treatment

Non-ITT Analysis				
**Status**	**Artesunate**	**Placebo**	**RD**	*p*-value*

Death ≤ 6 hours	56	51	-0.001	1.00
Death > 6 hours	98	126	0.005	0.14
Alive with PD	2	13	0.002	0.01
Alive and No PD	5916	5806	-0.006	-

Total	6072	5996		0.01^†^

**ITT Analysis**				

**Status**	**Artesunate**	**Placebo**	**RD**	*p*-value*

Death ≤ 6 hours	86	85	0.000	1.00
Death > 6 hours	172	203	0.004	0.26
Alive with PD	15	22	0.001	0.70
Alive and No PD	8681	8562	-0.004	-

Total	8954	8872		0.23^†^

Note: Late death (after 6 hours) assessed by day of the 7-30 day of follow up; *Multiple-comparison adjusted; ^†^Overall chi-square *p*-value.

We do this for two datasets. The top part of Table 5 shows the results for subgroup (iii), in line with the inappropriate ITT analyses of the authors. This shows a significant overall difference. Adjustment for multiplicity shows that this effect is driven by a difference in PD rates. The full ITT analysis is at the bottom of Table 5. In this, we assumed, as in Figure one of Gomes at al., that the eight lost to follow-up placebo-group cases were alive and without PD. This analysis points to a level of variation consistent with chance. Observe that when all the randomized subjects are considered, the PD rates for the two groups are nearly the same.

Lack of data prevented us from doing a complete ITT analysis of this form. Further, these analyses are tainted by the design and implementation problems noted above. These times to reach a referral clinic data are of too poor a quality to yield useful conclusions. Bearing these limitations in mind, we infer that a more comprehensive, unbiased, type I error protective analysis fails to back up the main conclusion on the utility of pre-referral artesunate suppository for presumed severe malaria drawn by Gomes at al.

Deciding upon ways of analyzing data after examining the data is a common but serious flaw in the analysis of clinical trial data [39]. Such data dredging increases the chance of generating spurious findings. Gomes et al. is replete with such practice. The *p*-values generated from such analyses lack rigorous probabilistic interpretation. Yet, they underlie the main conclusions reached by them. A better course of action is to fit a multi-variate regression model with pre-specified covariates and appropriate interaction terms.

#### Analysis and Reporting Metric

Gomes et al. use the risk ratio as the main comparative metric. When event rates are small (say, less than 5%), as is the case here, it may convey an exaggerated picture of the benefit of treatment. While there is debate among statisticians about the relative utility of the two metrics, we find arguments that the risk difference is preferred in such circumstances more persuasive [59,60]. The relative risk reduction for the main finding in the Summary of Gomes et al. is almost 50%, but the decrease in absolute risk is 1.9 percentage points. The former looks more impressive but is less useful in practical terms. This point has been made specifically for malaria interventions as well [61,62].

The number needed to treat (NNT) - the inverse of the risk difference - is a related metric. It is a suggested helpful metric for binary outcomes in the official explanatory document accompanying the CONSORT statement [14]. Gomes et al. do not report the NNT for the main outcomes but raise the issue in the discussion section. They state that they did not compute the NNT because baseline risks vary. As we noted earlier, the main data analyses of Gomes et al. were not stratified by center. Varied baseline risks were thereby not taken into account, and instead of weighted risk ratios, simple overall risk ratios were given. Their rationale for not computing the NNT is thus not consistent with doing the main analyses in an unstratified manner. If baseline risks are too variable to justify computing the NNT, that also calls for stratified analysis. Such analysis, further, is often warranted by design and effect measure heterogeneity considerations even when baseline risks do not vary.

Using the valid ITT numbers from the bottom half of Table 5, we find that the difference of risk for D or PD between rectal artesunate and placebo is 0.004 (*p *= 0.095) with 95% CI being (-0.001,0.010). This translates into an NNT of 250 with 95% CI equal to (NNTH 1000 to NNTB 10). For these ITT appropriate data, a risk ratio based analysis does not as well give a significant finding for this outcome. Of course, a better estimate of the NNT would come from a stratified ITT analysis for approximately fixed time window data. But the relevant data are not found from the paper.

#### Reporting Style

The vague or inconsistent manner of reporting the design and conduct of this trial were noted above. We also saw how non-primary endpoints became primary endpoints. Data analyses in the paper also shows examples of conceptual anomalies and reporting inconsistencies. For example, while the main analyses excluded those subjects with negative blood smears, for computing a risk difference, this subgroup is brought into the picture to argue that the risk difference is larger than what emerges from the computations. While stratification was not done in the main analysis, it was done in a subsidiary case; interaction testing for subgroup effects was not performed in general but was done for one particular analysis; survival analyses were not done but a curve appearing to depict it was presented; risk difference was not the main reporting metric but was employed as such in a subsidiary analysis; etc.

Figure one of Gomes et al., the patient flow chart, shows another instance of inappropriate reporting. Let us explain. Any clinical trial screens people deemed eligible but are, for some reasons, not enrolled. They may not have the disease in question, may refuse to participate, and so on. Such initial screening occurs in hospital-based and community-based trials. Often more people are excluded from a trial than included. The rural trial of Yeboah-Antwi et al. [31] had a total of 5108 children under five reporting to the recruiters. But only 3125 were enrolled. The numbers of cases excluded and the reasons for exclusions provide important indicators of the external validity of the trial. The patient flow chart is the main source for such information.

The flow chart of Gomes et al., Figure one, lacks such information, and gives the impression that everyone who approached the recruiters was randomized. But there are malaria cases with high fever who can take oral medication, or cases with fever and diarrhea not thought to be due to malaria. In such a large-sample study, it is not likely that there were no cases who came to the recruiters but were not randomized. It is more likely that such cases were not recorded or were not recorded consistently at all the centers. In the absence this crucial information about exclusions, this flow chart is a partially reported flow chart [13,14].

For clinical trials, the term pre-specified has a specific, narrow meaning. It refers to entities specified at the planning stage and noted in the protocol. This is how Gomes et al. use it most of the time. But when they refer to "*pre-specified exclusions*" of cases from data analysis at the beginning of the Results section, it has a different meaning, referring to being specified after the trial was over but before the results were unblinded for the final analysis.

There is no mention of interim analysis in Gomes et al. But from Kitua et al. [19] we learn that interim analyses were done but were not conclusive. We do not know if the exclusions from data analysis and the use of a composite outcome as a primary outcome in Gomes et al. were determined prior to or after these interim analyses. If it was the latter, an element of bias was introduced.

The reporting style in Gomes et al. overall does not facilitate illumination of the main results. Excessive space is given to secondary issues. Key matters appear in fine print. The reasoning is at times not clear. Information is scattered across different papers and sources. And what is said here is not always consistent with what is said there.

### Contextual Considerations

A clinical trial unfolds in a societal context. Apart from the therapies under scrutiny, context-specific factors usually affect the outcome of the disease at hand. For example, the prognosis of severe malaria in children in rural villages depends upon parental awareness; cost and promptness of care; transport services; availability of diagnostic tools, medication and hospital beds; quality of case management; and competency and motivation of health workers and hospital staff [19,63].

Protecting the welfare of people drawn into any phase of a clinical trial is essential. The trial design and conduct may modify some contextual factors to serve this end while the other factors are unchanged. Too many changes render the trial findings not applicable to that context. However, if even the gaping problems are left to fester, the welfare of the participants is undermined. Allowance for external validity has to balance concern for patient welfare. Contextual changes in a multi-center trial should reflect fairness across the centers and a common standard for patient welfare.

In this section, we examine the contextual interventions that Gomes et al. did or did not do. This allows us to judge the trial's external validity, and the degree of protection of patient welfare. We also discuss a contextual paradox that intruded into data analysis.

#### Contextual Interventions

The subjects in Gomes et al. encountered the trial-associated contextual changes at four occasions: (a) prior to enrollment; (b) at enrollment; (c) in transit to a referral clinic; and (d) at the referral clinic.

Before the trial start, the investigators held community meetings to educate and inform parents of potential participants. Specifically, "*the individual consent form, use of placebo, and the importance of proceeding to the referral clinic were discussed in detail and the trained local village recruiters were introduced*." (See also Kitua et al. [19]) This is as it should be. It is, however, not clear whether such a meeting was held at each of the 219 villages in the trial, and whether the meetings took place in a similar fashion at all the four centers.

At enrollment, the cases encountered the local recruiter. Using semi-trained workers in such a trial enhances the applicability of its results. Diseases with grave manifestations and outcomes, though, require VHWs with some prior training and sufficient experience to have developed the skills and fortitude to handle seriously ill children. VHWs need further training, as explained earlier. If pre-referral artesunate for suspected severe malaria is to be a real option, it will be implemented by VHWs. Persons with no health-related background and only a week of training are unlikely to be given such a responsibility [6].

Above, we noted the deficiencies and vagueness of Gomes et al. with regard to trainees and training, and contrasted it with Yeboah-Antwi et al. [31] The WHO/TDR website, however, declares that "*the training, supervision and monitoring of the resident village recruiters for the purpose of the clinical trial was more rigorous than that which would be the norm in many community settings*." [3] This compares training in the regular rural setting with that for an internationally funded trial. Such a comparison is unwarranted because not only does the latter have to abide by higher standards but it also has the resources to do so. We need to know if the training, supervision and monitoring in Gomes et al. trial was at par with or more rigorous than the norm for malaria or severe malaria trials in rural community settings. As noted earlier, such evidence has not been given. The recruitment of clearly ineligible cases, and the extent of missing data on key items we have detected do not favor it.

For transport to clinic, the level of support at different centers varied. In Tanzania, no support was given. In Ghana, a "*three-wheeled motorized transport was stationed at primary health centers to transfer patients to the district hospital*." [19] It is unclear if any arrangement for transport was made in Bangladesh.

Patients in Bangladesh, though, had stronger incentives to proceed with haste to the health center. Supplies of needed medications were stocked at the facilities in the area. Treatment and hospitalization were well organized and free of charge. In Ghana, that was not so. In Tanzania, where under the table payments for health services at governmental facilities are common, an unrealistic reliance was placed on the official policy that care would be provided free. Also, specific measures to ensure the supply of medicines were not taken. And, everywhere, "*[n]o change to the routine management of patients at hospital was made*." [19]

Variable contextual interventions contributed to varied levels of adherence to referral advice. Considering only the young children with malaria, in Tanzania, 18% never went to a clinic, while the same figures for Ghana and Bangladesh were 5% and 2%, respectively. The varied interventions lack a sound rationale. Severe malaria requires prompt appropriate care. Increasing support given to the parent raises the chance of the child's being taken to a clinic. The disparity among the centers reflects, in our view, lack of required coordination by the central organizing team. Or, it may be that these were three separate trials with distinct designs that which should have been published as three trials. Even in that case, a well argued rationale is needed for each trial.

The organizers knew of the possible dangers in aspects of the routine management of severe malaria [19]. In that respect, about 2000 of the 6000 patients who reached a referral clinic in Africa, mainly in Tanzania, and included in the analysis got intramuscular injections at an anatomically risky site. In eight cases, this caused serious damage to the sciatic nerve. Considering the cases excluded from the analysis, perhaps there were more. Adequate guidelines and supervision at the health clinics could have prevented this and other possible harm that we do not know about.

Inappropriate contextual intervention, especially the inadequate training of recruiters, perhaps had broader consequences. The rate of D or PD among those slide negative for malaria was 165/4648 (3.5%), among those already immediately treated by injection for severe malaria, it was 72/1110 (6.5%), and among (later) confirmed malaria cases, it was 346/12068 (2.9%) (chi-square *p *< 0.0001) (Figure one, Gomes et al.). The D or PD rate in cases with a prior injection was more than twice the rate of the last subgroup (*p *< 0.001, adjusted for multiple comparisons). The D or PD rate difference between these subgroups is also larger than the differences between artesunate and placebo highlighted by the authors. That is, the subgroups with either clearly ineligible or potentially ineligible cases had significantly higher D or PD rates. These higher rates may reflect the underlying risk in these subgroups, or they may be due to severely ill children being not handled appropriately, treated needlessly, and sent off unassisted on a hazardous trip when it was not called for. In any case, the claim that benefits of trial participation outweighed harm in these subgroups is subject to doubt. The variations in these subgroup level D or PD rates by center are not reported.

We note that the BMJ editorial [6] describing this trial as "*remarkable*" also devoted most of its space to argue that its findings may not be translatable into practice! The case was made on three grounds: (i) the inadvisability of setting up a parallel system (that is, outside of the usual VHW framework) to deliver rectal artesunate; (ii) the imperative, when a child is gravely ill, to ensure rapid referral to a facility that can give required treatment; and (iii) the question of how to deal with cases who show signs of severe malaria but may not have it. It thereby ends by praising Gomes et al. not for demonstrating that pre-referral rectal artesunate is a sound option in remote areas but for showing clearly that "*substantial delays in treatment can have serious effects on seriously ill children ...*" [6]. The latter, however, was neither the main aim of this trial, nor something that was backed with sound analysis by the authors.

The comment on Gomes et al. in *The Lancet *had called for field studies to address essential practical questions [2]. In the paper that addressed ethical issues, Kitua et al. [19], even the authors of Gomes et al. argue that the benefits of contextual interventions outweighed the difference between artesunate and placebo. To quote them: "*Because of rapid hospital referral, malaria mortality and morbidity decreased even without pre-referral treatment*."

Drug resistance is relevant. The strong discouragement of artemisinin monotherapy for uncomplicated malaria instituted by the WHO is meant to counter the emergence of resistant parasites. Gomes et al. also note the issue. Yet they do not pay due attention to what would happen if the rural areas are inundated with artesunate only pills. The attendant risks are many. In lay hands, they may be misused, overused and abused, and so hasten the reduction of the efficacy of a currently valuable remedy.

The WHO website, a response by the authors to a letter, and the paper on trial ethics by Kitua et al. stress that all the participants benefited from the conduct of this trial. They had better care and lower risk of death than would otherwise have been the case [3,19,64]. As we showed above, grounds for doubting this claim exist. Yet, even if it is valid, what does it say about the rationale for the trial?

A clinical trial is justified on the basis of an expectation of a meaningful difference in outcome, assurance of the welfare and rights of participants, a realistic assessment of eventual practical utility, good design and satisfactory implementation. It cannot be justified simply on the grounds of providing a benefit to the participants. Say, a rural randomized trial of severe malaria to compare a placebo suppository with a placebo skin ointment is planned. After the initial therapy, the child is sent to a health facility in an ambulance. Compared to a similarly afflicted child left in the village, this child gets better care and has improved chances of survival. That is true. Yet why spend millions of dollars on it - and multi-center international trials do cost millions of dollars - when that money can pay for better transportation for severely ill patients to health centers? If, furthermore, the trial has major problems in design, conduct, analysis and interpretation, the fact that all who took part in it benefited in some way does not serve to justify it.

Clinical trials must adhere to the basic ethical values of society. We do not plan a trial to test whether tobacco causes lung cancer. In the same fashion, we do not need clinical trials to improve access to care for severely ill children. An exclusive focus on clinical trials, particularly drug trials, fosters a myopic vision towards health care. Simpler and cheaper beneficial preventive or facilitatory measures are set aside in favor of treatments that have a marginal effect but which have been evaluated by randomized trials. In Africa this tendency has been carried to an extent that even the need for rural children to wash their faces has to be tested in an externally funded trial [65]. For internationally financed trials whose resources easily exceed the annual budgets of the health districts in which they take place, these are daunting concerns.

The very conduct of trials within some contexts can also generate a deleterious impact. Higher remuneration draws scarce health personnel into the trials to a degree that can compromise the care of regular patients. And public health priorities are misdirected as well. With more and more trials taking place in resource poor settings, these are not minor issues [66].

Contextual factors matter, and need careful attention when planning a trial. Else, even when it has been done according to high standards, its findings may be turn out to be superfluous. The BMJ editorial lauding this trial ends not with a call to promote rectal artesunate but with a call to fix the "*system failure*" that leads to delayed care [6]. The authors of Gomes at al. make the same point in another paper when they note that "*[h]ealth system improvements lowered the death rate*." [19] With its subtitle, "*Delays to treatment cost lives and quick fixes are not the answer*," the BMJ editorial [6] effectively poses the same question.

We have not come across a single paper that questions the major benefits of contextual interventions designed to enhance rapid referral. The shortfalls in such interventions and related matters we have noted therefore cast a negative shadow on the practical utility of Gomes et al.

#### A Contextual Paradox

The decision to include only confirmed cases of malaria in data analysis in Gomes et al. derived from a filing done with the US Food and Drug Administration (Statistical methods). It was, however, taken prior to unblinding the results. The FDA filing concerned cases with "*acute malaria*." But all the centers enrolled cases with "*suspected acute malaria ...*," or "*suspected severe malaria*," or a variation thereof (Methods part of the Summary). The party that did this filing is not named.

Rectal artesunate as a pre-referral remedy for severe malaria does not apply to the US context. There, if malaria or severe malaria is suspected, a blood slide will be read, and if positive, appropriate treatment administered promptly. The pre-referral option is called for in remote rural areas where severe malaria is suspected but not confirmed, and where, moreover, parenteral treatment is unavailable. By nature, it is a risk-reducing intermediate therapy based on presumptive diagnosis.

Restricting data analysis only to confirmed malaria cases then made the results appear less applicable to the rural areas of poor nations. This paradoxical deletion of about a quarter of subjects from analysis not only violated the ITT principle but compromised its external validity as well. That could have been avoided if all randomized subjects were included in the analysis. For the erroneously enrolled cases (with an immediate prior injection for malaria), the results of both including and excluding them in the analysis should have been presented. Also, note that we are unsure what proportion of the confirmed malaria cases fulfilled the WHO criteria for severe malaria.

It is unclear why this external factor - the filing to the FDA - was the main factor in deciding whom to exclude from data analysis. Gomes et al. is a scientific report, not an FDA application.

We end this section by clarifying that the term exclusion as used above does not imply practical neglect. Under a patient welfare protective design, cases presenting with severe symptoms and deemed eligible should be offered assistance to secure prompt treatment. But such help should also be given to the severe cases presenting to the recruiter but who are not deemed eligible for the trial. The latter, however, would not be enrolled in the trial or randomized. But their numbers and reasons for exclusion should be noted in the flow chart.

## Conclusions

This paper has shown that many facets of the study of Gomes et al. - design, conduct, analysis, interpretation and practical utility - had major flaws. The design flaws were manifested in the absence of well specified inclusion and exclusion criteria, outcome variables that were pre-specified, sample size computation, concrete time window for follow-up, and clear information on recruiters and training. Between center variability in number of blood slides, age of subjects enrolled, provision of transport and good care lacked a clear rationale. Shortfalls in implementation were evident through enrollment of clearly ineligible subjects and high missing levels for key data. The time window in the analysis mixed up short term and medium term mortality, and the survival analysis was not done. Excluding a third of the randomized cases from analysis not only violated the ITT principle but also compromised external validity, particularly since the exclusions stemmed from a contextually inappropriate factor. While the needed overall stratified analysis was not done, an excessive number of post-hoc subgroup analyses, many based on a post-randomization event, were done. An unbiased reanalysis of available trial data did not support the conclusions of the paper. The manner of reporting also left a lot to be desired. Essential information is publicly unavailable. Some information is internally contradictory. The contextual interventions, or lack thereof, did not protect patient welfare as it ought to have been protected, and because there was no clear rationale for their variability, compromised the applicability of the findings of the study.

Some of the problems we identified had a greater impact on the trial's scientific validity than others. Some introduced bias, some compromised external validity, some did both, and some were in the realm of reporting problems. It is generally agreed that a few trial features like adequacy of concealment of randomization, and the extent and pattern of missing data can have a major impact on scientific validity [67]. There is also agreement that poor statistical analysis can affect the validity and importance of the findings of a trial [68]. But a broader consensus about which problems have a major or greater impact on validity does not exist. For example, researchers differ in their judgment of the value of relative risk and risk difference as reporting metrics.

For Gomes at al., we do not list the problems in order of importance. Many of them are interrelated. Further, we focus on the assessment that emerges from viewing them in their totality. We hold that taken as whole, the problems we uncovered severely compromise the scientific validity of the trial, and the applicability of its findings.

The comment in the *Lancet *on this trial declared: "*If there are a handful of important papers every decade that will influence the way malaria is treated, this is one of them*." [2] Based on what we have shown, we firmly disagree with this characterization. On the contrary, we deem Gomes et al. as an extensively flawed trial whose conclusions and practical applicability remain subject to appreciable doubt.

It is sobering to note that problems of this scope and magnitude occurred even though the trial had a two-year preparatory and planning phase, noted authorities in the field were involved, it was coordinated by a globally esteemed institution in tropical diseases research, and was overseen by a reputable clinical trial center with leading experts and long experience in trial design, analysis and reporting. One of their key tasks was to harmonize the design features between the trial centers. That could have been done better.

Historically, an excess of poor quality trials was associated with an excessive number of small size trials. In some fields, large size trials have generally been better quality trials though this relationship between trial quality and size does not always hold [69,70]. Yet, a large multi-center trial with adequate resources and expertise is an opportunity to produce reliable findings, set a high methodological standard, and even introduce methodological innovation [54].

Gomes at al. represents the culmination of a ten year effort by dedicated researchers with thousands of participants on the ground. The quality of the final product is then all the more distressing to contemplate. A stupendous opportunity to gather reliable information to enhance public health that does not arise often was regrettably missed.

Our verdict on this trial has some general implications. First, poor quality trials continue to appear even in reputable medical journals [71,72]. This trial appeared in a premier journal which subscribes to the current standards for quality and requires trial authors to adhere to the CONSORT statement. Yet, a paper about a major health issue but with grave flaws not only got into print, but also went on to secure high praise and win a major award. Some flaws could have been easily detected if the CONSORT checklist was applied. Our paper adds to the series of cases of recent papers the scientific community failed to review adequately prior to publication. It thus underscores the need to continually scrutinize and improve the peer review process [73]. The journals in question may also need to examine the process by which this paper was reviewed.

Second, we stress that in this era of electronic publishing - when journals and organizations put supplementary material on web pages - and of greater awareness about the need for transparency, lack of space is not a valid reason for the absence of critical information. Note also that the information about sample size calculation and eligibility criteria for this trial would have taken much less space as compared to the extensive space the authors gave to subsidiary secondary analyses.

Third, our paper underscores the proposition that assessing trial quality from a checklist is not sufficient to obtain a valid judgment of its quality [74]. A trial can fulfill all or most of the checklist criteria for a good quality trial. Yet, it can be deeply flawed. And, in some cases, the situation may perhaps be the other way around. Well designed and validated checklists are needed, but they should be supplemented with an in-depth evaluation.

Fourth, ITT analysis is a valuable tool to protect the control of bias achieved through randomization, avoid attrition and other biases, and enhance external validity. Yet, it continues to be misinterpreted and misutilized [75-77]. An analysis based on removal of a third of randomized subjects from a large trial that is still labeled an ITT based analysis represents an extreme case of this. Also, despite the numerous cautions given about subgroup analysis, it continues to prevail. Health and medical journals need to do more to bring practice in line with principles.

Fifth, the results of Gomes et al., the various commentaries on it, and associated papers as well as our paper point to the importance of considering contextual factors when planning a trial. There is, in particular, a critical need in most poor nations for regular general health education via the mass media, public meetings and adult education campaigns. Yet, this matter has had a low priority in the recent years. When external funds permit, messages on HIV/AIDS or malaria are heard on the radio. While enticing promotions of soft drinks and chewing gum permeate the air in the most far away areas, comprehensive education on health, hygiene and nutrition is all but absent. Health literacy is generally at a low level and people harbor all manner of beliefs relating to health [78]. A systematic review of qualitative malaria studies identified several barriers to effective prevention and treatment, one being the belief that a child who is having convulsions may "*die if given an injection or taken to a hospital*." [79] For introduction of rectal artesunate therapy, Kaona and Tuba [80] suggest effective prior sensitization among mothers and health workers.

Promoting prompt treatment for seriously ill children should not await the conduct of a clinical trial. Rather, it should be an ongoing educational activity done effectively with local funds. Correspondingly, the educational effort of the type done by Gomes et al. to improve adherence to referral must be a part of a wider process of system change noted by the *BMJ *editorial [6]. Education done specifically for a clinical trial may have a selective focus, and thus be of questionable long term utility.

Lack of access to the original data is a major limitation of our paper. An independent evaluation of detailed trial records is thereby in order. A reanalysis of the data of Gomes et al. using appropriate methods should follow. Only in that way can more well founded conclusions be drawn. The contextual concerns we have raised need attention as well.

We end by emphasizing that our paper does not take a position on the use or otherwise of rectal artesunate as a pre-referral treatment for suspected severe malaria. Our stand is that this question is too important to be decided from an inadequately designed, poorly conducted, erroneously analyzed, and selectively interpreted study. The welfare of the children and adults with or at risk for severe malaria can only be served by trials that are meticulously planned, performed according to sound scientific principles, and analyzed appropriately, and whose contextual ramifications are unimpeachable. The recommendation on rectal artesunate as a pre-referral treatment for suspected severe malaria needs to await the results of such a trial.

At the same time, we must ponder: Should such a trial be carried out or should the focus be on contextual changes to promote health education and speedier access to treatment? The latter benefits not just the cases with severe malaria but many more who require urgent attention. The question is: Should the future research and policy agenda derive from the endorsement of rectal artesunate as a pre-referral in the Summary of Gomes et al? Or should it derive from the last sentence of the same paper, which declares that "*accessible clinics and good organization within villages and within hospitals can greatly reduce malaria mortality and morbidity even without pre-referral treatment*." The emergent agendas, one directed towards development of improved pre-referral suppositories, [81] and the other towards "*addressing access barriers among the poor and the vulnerable*," [82] are quite divergent.

## Abbreviations

CI: confidence interval; D: death; ITT: intent-to-treat; NNT: number needed to treat; PD: permanent disability; RD: risk difference; TDR: UNICEF/UNDP/World Bank/WHO Special Programme for Research and Training in Tropical Diseases; VHW: village health worker.

## Competing interests

ZGP has been a paid consultant for, and received research funding from pharmaceutical companies involved in malaria research. In relation to this study, KFH and ZGP have no competing interests.

## Authors' contributions

KFH: Conceived and designed the study, collected and analyzed the data, wrote all drafts of the paper. ZGP: Provided comments and additional material. (KFH contributed 90% and ZGP contributed 10% of the total effort). KFH, ZGP: Read final draft and agreed with the results and conclusions.
